# Mental illness rates among employees with fixed-term versus permanent employment contracts: a Danish cohort study

**DOI:** 10.1007/s00420-022-01936-7

**Published:** 2022-11-22

**Authors:** Harald Hannerz, Hermann Burr, Martin Lindhardt Nielsen, Anne Helene Garde, Mari-Ann Flyvholm

**Affiliations:** 1 National Research Centre for the Working Environment, Lersø Parkallé 105, 2100 Copenhagen, Denmark; 2 Federal Institute for Occupational Safety and Health, BAuA, Nöldnerstr. 40–42, 10317 Berlin, Germany; 3 Lægekonsulenten.dk, Hasselager Centerue 35, 8260 Viby J, Denmark; 4 Department of Public Health, University of Copenhagen, 1014 Copenhagen, Denmark

**Keywords:** Cohort study, Fixed-term employment, Permanent employment, Psychotropic drugs, Psychiatric hospital treatment

## Abstract

**Purpose:**

It has been hypothesized that employment in a fixed-term instead of permanent contract position is associated with an increased risk of development of mental health problems. The present study aimed at estimating rate ratios between fixed-term and permanent employees in the Danish labor force, for use of psychotropic drugs and psychiatric hospital treatment due to mood, anxiety or stress-related disorders, respectively.

**Methods:**

Employment data were drawn from the Danish Labor Force Survey of 2001–2013, which is a part of the European Labor Force Survey. Full-time employed survey participants without mental illness at the baseline interview (*N* = 106,501) were followed in national health registers for up to 5 years. Poisson regressions were used to estimate rate ratios for redeemed prescriptions of psychotropic drugs and psychiatric hospital treatments due to mood, anxiety or stress-related disease. The analyses were controlled for age, gender, industrial sector, nighttime work, level of education, calendar year, disposable family income and social transfer payments within 1 year prior to the baseline interview.

**Results:**

The rate ratio for hospital diagnosed mood, anxiety or stress-related disorders among employees with fixed-term vs. permanent employment contracts was estimated at 1.39 (99.5% CI 1.04–1.86), while the corresponding rate ratio for redeemed prescriptions of psychotropic drugs was estimated at 1.12 (99.5% CI 1.01–1.24).

**Conclusion:**

The present study supports the hypothesis that employment in a fixed-term rather than permanent contract position is associated with an increased risk of developing mental health problems.

**International registered report identifier (IRRID):**

DERR2-10.2196/24392.

**Supplementary Information:**

The online version contains supplementary material available at 10.1007/s00420-022-01936-7.

## Introduction

It has been hypothesized that fixed-term contract workers are at higher risk of developing mental health problems than permanently employed workers. A reason for this hypothesis is that the job insecurity associated with a non-permanent employment position may act as a stressor that may induce fears and worries about future unemployment, which in turn may increase a person’s vulnerability to mental ill health (Rönnblad et al. 2019). Another reason for believing in a prospective association between fixed-term contract positions and the risk of developing mental ill health is that the expiry date of a fixed-term employment contract may be followed by a spell of involuntary unemployment, which is a well-established risk factor of mental ill health (Paul and Moser 2009). The relationship between perceived job insecurity and subsequent mental ill health is well established. A recent review and meta-analysis of longitudinal studies (Rönnblad et al. 2019) estimated the odds ratio (OR) for adverse mental health among workers with self-reported job insecurity compared with workers without self-reported job insecurity at 1.52 [95% confidence interval (CI) 1.35–1.70]. The meta-analysis included 14 studies, with a total number of 43 568 participants. The OR was greater than one in all of the included studies. The same review article could, however, not establish a relationship between objective indicators of job insecurity, i.e., specific types of employments, and mental ill health. In particular, it could not establish that employment in a fixed-term instead of permanent contract position was associated with an increased risk of mental ill health; only a few such studies had sufficient quality, and their results were inconsistent (Rönnblad et al. 2019).

Four longitudinal studies, one German (*n* = 2009), one Swedish (*n* = 660) and two Finnish (*N* = 65,208 and *n* = 107,828), have estimated longitudinal associations between fixed-term vs permanent employment and indicators of mental ill health (Hammarström et al. 2011; Virtanen et al. 2008; Ervasti et al. 2014; Demiral et al 2022). Only the German study was on a representative employee population, the two Finnish were on public sector employees and the Swedish study was on a follow-up of ninth-grade graduates. So we know little regarding the possible mental health effects of fixed-term contracts in representative employee populations.

The effects of fixed-term contracts on health might be dependent on welfare state type. The Danish flexicurity welfare state type, characterized by low employment protection, high compensation for unemployed—even if the level of compensation has decreased somewhat—and a high turnover (Madsen 2006, 2013). This has led to a relatively low fraction of long-term unemployed among the unemployed and a low overall experience of job insecurity (Madsen 2006). One recent international comparative analysis shows that—in Denmark—the level of employment protection is generally low both among fixed-term contracts and open-end contracts—a combination which has been found to be beneficial for general health among those in fixed-term contracts (Voßemer et al. 2018). This comparative study, however, indicates that findings even from other North European countries, such as those mentioned above, cannot be transferred to a Danish context.

Also, it should be noted that perceived job insecurity is a subjective construct that may be influenced by an individual’s personality traits. It has, for example, been shown that neuroticism is associated with perceived job insecurity (Blackmore and Kuntz 2011) as well as mental ill health (Lahey 2009). Hence, until it has been established that the prospective association between job insecurity and mental ill health also holds good for objective indicators of job insecurity, we cannot rule out the possibility that the positive association between self-reported job insecurity and development of mental ill health only appears among questionnaire respondents with, e.g., varying degrees of neuroticism.

The present study aimed at estimating rate ratios between fixed-term and permanent employees in the Danish labor force, for use of psychotropic drugs and psychiatric hospital treatment due to mood, anxiety or stress-related disorders, respectively. The present study would thereby contribute to the international literature with information on relative rates of mental ill health between fixed-term and permanent employees in a nation with generous unemployment benefits and a legislation, which protects fixed-term contract workers against discrimination at the work place (The Council of the European Union, 1999).

## Methods

The data material and statistical methods of the present study were completely specified, peer reviewed and published in a study protocol (Hannerz et al. 2021) before we looked for any relation between the exposure and the outcome data of the study. The protocol defines two separate studies. One of the studies would compare rates for use of psychotropic medicine and psychiatric hospital treatment among fixed-termed versus permanently employed people, while the other would do the same thing for fixed-termed employed versus unemployed people. The former of these studies is reported in the present paper, while the latter will be reported elsewhere.

The study protocol contains the following copyright and license information: “©Harald Hannerz, Hermann Burr, Helle Soll-Johanning, Martin Lindhardt Nielsen, Anne Helene Garde, Mari-Ann Flyvholm. Originally published in JMIR Research Protocols (http://www.researchprotocols.org), 05.02.2021. This is an open-access article distributed under the terms of the Creative Commons Attribution License (https://creativecommons.org/licenses/by/4.0/), which permits unrestricted use, distribution, and reproduction in any medium, provided the original work, first published in JMIR Research Protocols, is properly cited.”

Relevant methodological details from the study protocol will be repeated or paraphrased in the method section of the present paper.

### Data material

This study was based on baseline data on employment status from the Danish Labor Force Survey (DLFS) (Statistics Denmark 2019), which is the Danish part of the European Labor Force Survey (Eurostat 2021). Data from 2001 to 2013 were used for baseline, and follow-up data on health came from a series of registers, which cover the entire population of Denmark. The following registers were used: the Central Person Register (CPR) (Pedersen 2011), the Employment Classification Module (ECM) (Petersson et al. 2011), the Danish Education Registers (Jensen and Rasmussen 2011), the Danish Family Income Register (Statistics Denmark 2020), the Danish Register for Evaluation of Marginalization (DREAM) (The Danish Agency for Labour Market and Recruitment 2019), the Psychiatric Central Research Register (Mors et al. 2011), and the National Prescription Register (Kildemoes et al. 2011). Linkage on an individual level was based on participants’ personal identification numbers (Pedersen 2011).

DLFS is based on quarterly random samples of 15- to 74-year-old residents of Denmark, with systematic oversampling of unemployed people. Each participant is invited to be interviewed four times over the course of a year-and-a-half. The purpose of the interviews is to collect person-based information on inter alia, labor market attachment, type of contract, and working hours (Statistics Denmark 2019; Eurostat 2021). Among those invited for the DFLS, the response rate decreased over time from 70% in 2002 to 53% in 2013 (Hannerz et al. 2018). The CPR contains, inter alia, information on gender and dates of birth, death, and migrations for every person who is or has been a resident of Denmark sometime between 1968 and the present time. The ECM contains annual, person-based information on, inter alia, the socioeconomic status, occupation, and industry of the residents of Denmark. The Danish Education Registers contain person-based information on, inter alia, a person’s highest educational attainment. The Danish Family Income Register contains information on household income. DREAM contains weekly, person-based information on social transfer payments (welfare benefits payments) such as maternity and paternity benefits, sickness absence benefits, unemployment benefits, social security cash benefits, and state educational grants. DREAM has existed since 1991 and covers all residents of Denmark. The weekly benefits data are recorded if the person has been on a benefit for 1 or more days of the week. However, as only one type of social transfer payment can be registered per week, types of benefits are prioritized in the case of data overlap. The above-mentioned social transfer payments are prioritized in the order listed, that is, maternity and paternity benefits have higher priority than sickness absence benefits, which in turn have higher priority than unemployment benefits, etc. The Psychiatric Central Research Register contains person-based information on inpatients, outpatients, and emergency ward visits in all psychiatric hospital departments in Denmark. The National Prescription Register contains person-based data on all redeemed prescriptions at pharmacies in Denmark.

### Clinical end points

Rate ratios were examined for the following end points:

- Redeemed prescriptions for any type of psychotropic medicine, that is, drugs in the ATC-code category N05 (psycholeptica) or N06 (psychoanaleptica)
- Psychiatric hospital treatment with mood, anxiety, or stress-related disorder (ICD-10: F30–F41 or F43) as the principal diagnosis

The following mental disorders are included in the above case definition:

- F30 Manic episode
- F31 Bipolar affective disorder
- F32 Depressive episode
- F33 Recurrent depressive disorder
- F34 Persistent mood (affective) disorders
- F38 Other mood (affective) disorders
- F39 Unspecified mood (affective) disorder
- F40 Phobic anxiety disorders
- F41 Other anxiety disorders
- F43 Reaction to severe stress and adjustment disorders

### Exposure

The participants were categorized as “employed on a fixed-term contract position” or “employed on a permanent contract” in accordance with their responses to the question “Do you have a temporary or permanent employment contract?”

### Control variables

The analyses were controlled for gender, age (10 year classes), calendar year of the interview (2001–2003, 2004–2006, 2007–2009, 2010–2013), disposable family income (tertiles), educational level (low, medium, high, unstated), industry (“agriculture, forestry, hunting, and fishing”, “manufacturing, mining, and quarrying”, “construction”, “wholesale, retail and repair of motor vehicles”, “transporting and storage”, “accommodation and food service activities”, “human health and social work activities”, “other industries”, “unstated”), nighttime work (regularly, occasionally, never) and reception of maternity or paternity benefits (yes, no), unemployment benefits (yes, no) and state educational grants (yes, no) sometime during the 1-year period preceding the baseline interview. The variables “gender”, and “age”, refer to the status at the time of the baseline interview. The variables “disposable family income”, “educational level” and “industry group”, refer to the status in the calendar year preceding the interview. The variable “nighttime work” refers to a 4-week period preceding the interview. Further details about the operationalization of the control variables are given in our study protocol (Hannerz et al. 2021).

### Follow-up

The follow-up in the register data started on the date when 6 weeks had passed since the first DLFS interview and ended on the date when any of the following events occurred: the participant emigrated, the participant died, the participant met the clinical end point of the analysis, 5 years had passed since the date of the start of the follow-up, or the study period ended. The end of the study period was set at the end of the calendar year 2014 for redeemed prescriptions of psychotropic drugs and 2017 for psychiatric hospital treatments. Person-years at risk were calculated for each of the included participants. Participants who died or emigrated during the follow-up were censored at the time of the event.

The present study had access to the data on Anatomical Therapeutic Chemical Classification System (ATC) codes from the National Prescription Register for the time period 2000–2014 and International Statistical Classification of Diseases and Related Health Problems, 10th Revision (ICD-10) codes from the Psychiatric Central Research Register for the time period 1995–2017. Thus, the follow-up periods regarding the two outcomes differed in length.

### Inclusion criteria

The primary analyses were based on data from the participants’ first interview in the time period 2001–2013. Participants were eligible for inclusion if the following criteria were fulfilled:

1. The participants were aged between 20 and 59 years at the time of the interview.
2. They were employed, according to the interview.
3. They usually worked ≥ 32 h a week, according to the interview.
4. They did not receive any social transfer payments (other than holiday allowance, unemployment benefits, maternity/paternity benefits, or state educational grants) during the 1-year period preceding the interview.
5. They did not receive any psychiatric hospital treatment with mental disorders (ICD-10: F00–F99) as the principal diagnosis during the 1-year period preceding the start of follow-up.
6. They did not redeem any prescription for psychotropic drugs (ATC: N05–N06) during the 1-year period preceding the start of follow-up.

Since the fulfillment of inclusion criteria 4–6 only could be ascertained for participants who lived in Denmark throughout the 1-year period preceding baseline, we excluded all participants who migrated within this period. We also excluded participants with missing values on the covariates of the analysis. The reason for excluding part-time workers is that some workers may have chosen to work part time due to ill health. Based on survey data (cf. Feveile et al., 2007), we estimated that part-time workers who also had a fixed-term contract constituted approximately 1.5% of all employees in 2005.

### Primary statistical analysis

Poisson regression was used to estimate RRs for psychiatric hospital treatment for mood, anxiety, or stress-related disorders and redeemed prescriptions for psychotropic drugs, as a function of employment status at baseline (full-time fixed-term contract versus full-time permanent contract). The analyses were adjusted for age, gender, disposable family income, educational level, calendar year of the interview, baseline industry group, nighttime work and reception of maternity or paternity benefits, unemployment benefits and state educational grants sometime during a 1-year period preceding baseline. The logarithm of person-years at risk was used as an offset. Likelihood ratio tests were used to test first for main effects and then for effects of interaction with gender, age, and education level. The main effects were tested both for psychiatric hospital treatments and redeemed prescriptions for psychotropic drugs. Due to power concerns, the interaction effects were only tested for redeemed prescriptions for psychotropic drugs. Each of the tests were conducted at the significance level 0.005.

We controlled for industry, as a previous study has found significant industry-related inequalities in the rate of mood disorders among employees in the general working population of Denmark (Hannerz et al. 2009). We controlled for unemployment benefits and state educational grants in the 1-year period preceding the interview, as we believe that people’s attitudes toward fixed-term and permanent contracts may depend on their previous labor market attachment. We controlled for nighttime work because it has been shown that the prevalence of psychotropic drug usage in Denmark is greater among shift workers than among workers without shift work (Albertsen et al. 2020). We controlled for reception of maternity or paternity benefits, since the birth of a child may result in maternal (O'Hara and McCabe 2013) and paternal (Scarff 2019) postpartum depression. The remaining control variables were included, since the literature suggests that the risk of mental ill health depends on gender (Parker and Brotchie 2010; McLean et al. 2011), age (Wittchen and Hoyer 2001; Tjepkema 2005; Kessler et al. 2010), calendar year (Steinhausen and Bisgaard 2014), education level (Andrade et al. 2000), and income (Orpana et al. 2009; Schlax et al. 2019; Kosidou et al. 2011; Patel et al. 2018).

We tested for interactions, as it has been suggested that the strength of adverse health effects of fixed-term contracts depends on gender (Pirani and Salvini 2015), age (Wanberg et al. 2016), and education level (Virtanen et al. 2008).

### Sensitivity analyses

A series of pre-specified sensitivity analyses were conducted to: (i) estimate rate ratios in a subset of the study population where exposure is more stable over time, (ii) estimate rate ratios without control for industrial sector, nighttime work, calendar year, disposable family income and welfare benefits within the 1-year prior to baseline, (iii) estimate rate ratios by industrial sector, (iv) compare rate ratios obtained with and without exclusion of former cases of psychiatric treatment, (v) examine rates as a function of reason for having a fixed-term employment contract, and (vi) estimate relapse rate ratios.

The motivations, methods and results of the sensitivity analyses are presented in the appendix.

## Results

**Fig. 1:**
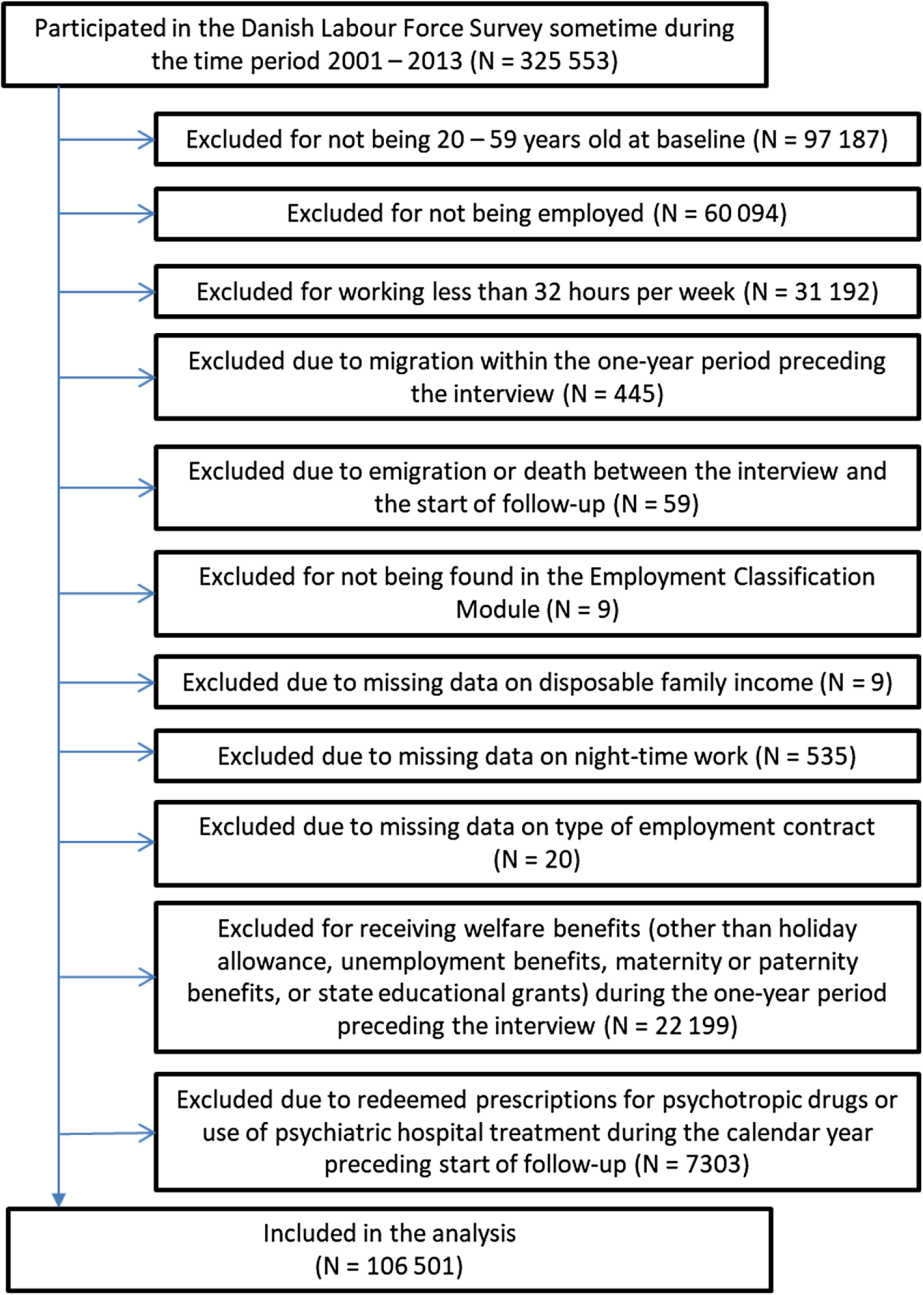
Flowchart for inclusions and exclusions of the primary analysis

Between 1 January 2001 and 31 December 2013, 325 553 persons participated in the DLFS, whereof 106 501 were eligible for inclusion in the primary analysis of the present study. A flowchart for inclusions and exclusions of the primary analysis is given in Fig. 1.

Among the included participants, we detected 11 616 cases of redeemed prescriptions for psychotropic drugs and 948 cases of mood, anxiety or stress-related psychiatric hospital treatment, in 430 733 and 519 162 person-years at risk, respectively.

Among the cases of psychiatric hospital treatment, 0.6% were manic episodes (ICD-10: F30); 2.0% were bipolar affective disorders (F31); 27.5% were depressive episodes (F32); 8.9% were recurrent depressive episodes (F33); 0.7% consisted of persistent (F34) other (F38) or unspecified affective mood disorders (F39); 2.5% were phobic anxiety disorders (F40); 9.0% were other anxiety diagnoses (F41); and 48.7% consisted of adjustment disorders and reactions to severe stress and (F43).

Among the cases of psychotropic drug use, 2.6% of the prescribed drugs were antipsychotics (ATC-code: N05A); 21.9% were anxiolytics (N05B); 35.8% consisted of hypnotics and sedatives (N05C); 39.1% were antidepressants (N06A); 0.57% were psychostimulants (N06B); 0.00% were antidepressants in combination with psycholeptics (N06C); and 0.05% were antidementia drugs (N06D).

The rate ratio for hospital diagnosed mood, anxiety or stress-related disorders among employees with fixed-term vs. permanent employment contracts was estimated at 1.39 (99.5% CI 1.04–1.86), while the corresponding rate ratio for redeemed prescriptions of psychotropic drugs was estimated at 1.12 (99.5% CI 1.01–1.24). The rate ratios for redeemed prescriptions of psychotropic drugs seemed to be statistically independent of age, gender and education level; the *P* values of the tests for interaction were estimated at 0.62, 0.23 and 0.22, respectively. The statistical power was too low to allow testing for interaction effects for psychiatric hospital treatment.

The rate ratios, number of persons and person-years at risk in the analyses of redeemed prescriptions of psychotropic drugs are given in Table 1, with and without stratification by gender, age and education level. The results of the hospital treatment analysis are given in Table 2.

**Table 1:** Rate ratio (RR) with 99.5% confidence interval (CI) for use of psychotropic drugs, as a function of type of employment contract among full-time employees in Denmark 2001–2013

Type of population		Type of employment contract	Persons	Person-years	Cases	RR	99.5% CI
All employees^a^		Fixed-term	7460	29,242	903	1.12	1.01–1.24
		Permanent	99,041	401,491	10,713	1	–
Gender strata^b^	Men	Fixed-term	3400	13,162	323	1.18	1.00–1.40
		Permanent	54,590	223,737	4843	1	–
	Women	Fixed-term	4060	16,079	580	1.08	0.95–1.23
		Permanent	44,451	177,754	5870	1	–
Age strata^c^	20–29 years	Fixed-term	3522	13,490	284	1.05	0.87–1.27
		Permanent	13,222	54,039	1043	1	–
	30–39 years	Fixed-term	1814	7171	246	1.18	0.97—1.43
		Permanent	26,526	109,248	2615	1	–
	40–49 years	Fixed-term	1056	4277	171	1.10	0.88–1.38
		Permanent	30,547	122,505	3469	1	–
	50–59 years	Fixed-term	1068	4304	202	1.16	0.94–1.44
		Permanent	28,746	115,698	3586	1	–
Educational level strata^d^	High	Fixed-term	2279	8710	259	1.10	0.92–1.33
		Permanent	30,425	120,019	3090	1	–
	Medium	Fixed-term	3208	12,855	404	1.15	0.99–1.34
		Permanent	51,442	210,732	5374	1	–
	Low	Fixed-term	1908	7498	228	1.06	0.86–1.29
		Permanent	16,393	67,850	2154	1	–
	Unstated	Fixed-term	65	179	12	2.05	0.87–4.86
		Permanent	781	2890	95	1	–

^a^Adjusted for age, gender, industrial sector, nighttime work, education, calendar year, disposable family income and state educational grants, unemployment benefits and maternity/paternity benefits within 1 year prior to baseline

^b^Adjusted for all of the above control variables except gender

^c^Adjusted for all control variables except age

^d^Adjusted for all control variables except education level

**Table 2:** Rate ratio (RR) with 99.5% confidence interval (CI) for psychiatric hospital treatment due to mood, anxiety or stress-related disorders, as a function of type of employment contract among full-time employees in Denmark 2001–2013

Type of employment contract	Persons	Person-years	Cases	RR^a^	99.5% CI
Fixed-term	7460	35,635	132	1.39	1.04–1.86
Permanent	99,041	483,527	816	1	–

^a^Adjusted for age, gender, industrial sector, nighttime work, education, calendar year, disposable family income and state educational grants, unemployment benefits and maternity/paternity benefits within 1 year prior to baseline

None of the results obtained in the sensitivity analyses were drastic enough to invalidate the findings of the primary analyses [cf. Appendix: Tables S1–S6]. The sensitivity analysis, which stratified rate ratios for use of psychotropic drugs by industrial sector, suggested, however, that the effect of having a fixed-term versus permanent employment contract may be especially high in the transport and storage industry, where the rate ratio was estimated at 1.87 (99.5% CI 1.14–3.07) [cf. Appendix: Table S6].

Apart from the pre-specified sensitivity analyses, we conducted two post hoc sensitivity analyses. In one of the analyses, we excluded phobic anxiety disorders from the case definition. All other details of the analysis were the same as in the primary analysis of the psychiatric hospital treatments. In this post hoc sensitivity analysis, the concerned rate ratio was estimated at 1.42 (99.5% CI 1.06–1.91). In the other post hoc analyses, we extended the required period of “no psychiatric hospital treatment” from 1 to 5 years prior to the baseline interview. All other details of the analysis were the same as in the primary analysis of the psychiatric hospital treatments. In this post hoc sensitivity analysis, the concerned rate ratio was estimated at 1.34 (99.5% CI 0.98–1.82).

## Discussion

### Main findings

We found that the rate ratios for use of psychotropic drugs and psychiatric hospital treatment due to mood, anxiety or stress-related disease, in the Danish labor force, were statistically significantly higher among employees with fixed-term vs. permanent employment contracts. The tests for interactions with age, gender and education level were not statistically significant.

### Results in relation to previous research

We found four relevant studies that estimated longitudinal associations between fixed-term vs permanent employment and indicators of mental ill health, one from Germany (Demiral et al. 2022), one from Sweden (Hammarström et al. 2011) and two from Finland (Virtanen et al. 2008; Ervasti et al. 2014).

The German study dealt with employees in employments subject to social security payments (Demiral et al. 2022) aged 31–60 years—representing 80% of all people working in that age range (*n* = 2009). Odds ratios for depressive symptoms as a function of fixed-term employment contract (yes vs. no) were 2.20 (95% CI 0.80–6.06) among men and 1.42 (0.61–3.32) among women. The analyses were adjusted for baseline (2012) age, partnership status and socioeconomic position. The study population of the Swedish study (Hammarström et al. 2011) consisted of all ninth-grade graduates of the calendar year 1981, in Luleå, who held temporary and/or permanent employment contracts between the age of 30 and 42 years (*n* = 660). Questionnaire data were collected at the age of 30 and 42 years. Odds ratios at the age of 42, for the contrast “temporary employment for a total time of more than 10 months” versus “permanent employment during the whole 12-year period” were estimated at 1.90 (95% CI 1.33–2.71) for psychological distress and 1.79 (95% CI 1.04–3.08) for depressive symptoms. The analyses were controlled for gender, self-rated health, psychological distress and depressive symptoms at age 30.

One of the Finnish studies (Virtanen et al. 2008) examined associations between temporary employment and redeemed prescriptions for antidepressant medication (1998–2002) among 17,071 men and 48,137 women employed municipalities in Finland. After adjustment for age, socioeconomic status (SES), and calendar year, the odds ratio for the contrast “fixed-term > 6 months vs. permanent employment” was estimated at 1.18 (95% CI 1.03–1.37) for antidepressant use in men and 0.99 (95% CI 0.93–1.06) in women. The corresponding odds ratios for the contrast “fixed-term <  = 6 months vs. permanent employment” were estimated at 1.43 (95% CI 1.19–1.73) in men and 1.18 (95% CI 1.09–1.28) in women, and for the contrast “subsidized temporary work vs. permanent employment” they were estimated at 1.57 (95% CI 1.23–2.02) in men and 1.38 (95% CI 1.20–1.59) in women. The association between type of employment contract and use of antidepressants was statistically significantly weaker among women than it was among men (*p* = 0.007). The association was, moreover, weaker among men with high SES than it was among men with low SES (*p* = 0.033).

The other Finnish study (Ervasti et al. 2014) examined associations (2005–2011) between temporary vs. permanent employment and sickness absence due to medically certified depressive disorders (ICD-10 codes F32–F34) among 107,828 Finnish public sector employees. The concerned rate ratio was estimated at 1.02 (95% CI 0.97–1.08). The analysis was adjusted for age, gender, level of education, chronic somatic disease and history of work disability due to mental or behavioral disorder (ICD-10 codes F00–F99). No significant interaction with gender, age, or education was observed (*p* > 0.25).

The associations between fixed-term contracts and mental ill health that were observed in the present study aligns well with the findings of the Swedish study (Hammarström et al. 2011) and the first of Finnish studies (Virtanen et al. 2008). The German study’s relatively small population size combined with low prevalence of fixed-term contracts might explain its insignificant findings (Demiral et al. 2022). A possible explanation for the null-finding observed in the second of the Finnish studies (Ervasti et al. 2014) is that it did not estimate rate ratios for depressive disorders but for sickness absence due to depressive disorders. Some workers with depressive disorders may call in sick while others may continue to work, and it has been shown that temporary employees, due to job insecurity, tend to have higher rates of sickness presence than permanent employees do (Virtanen et al. 2003; Reuter et al. 2019). It might also be that different levels of employment protection in fixed-term contract and in permanent contracts across countries could lead to country dependent associations between contract type and health (Voßemer et al. 2018).

### Methodological considerations

Our study has several strengths. The study was quite large and the statistical power was high enough to investigate main effects of having a fixed-term versus permanent employment contract. Bias from missing follow-up data was substantially reduced, since the endpoints of the study were ascertained through national registers that cover all inhabitants of the target population. Within-study selection bias was eliminated, since all hypotheses, significance criteria, endpoints and statistical methods were completely defined and published before we looked at any relation between the exposure and outcome data of the study (Hannerz et al. 2021).

The major drawback of the study is that it is observational and thereby has a weaker design than a randomized controlled trial, which is the golden standard in determining causality. Another weakness is the low response rate, which means that we cannot rule out the possibility of non-response bias. We believe, however, that any such bias has been mitigated by the many control variables that were included in the analyses. Individual participant data were available on a large variety of socioeconomic and occupational factors, which enabled us to control the analyses for a series of possible confounders and health selection effects such as age, gender, education, industry, nighttime work, unemployment benefits and income. Control for unemployment is relevant in order to take selection into part-time work into account. Control for income is important, as it has been found that effects of insecurity in employment can be alleviated by increased wage levels (Böckerman et al. 2011).

It has been shown that the risk of developing depression is associated with smoking habits (Pasco et al. 2008; Korhonen et al. 2007) and body mass index (Luppino et al. 2010). In the present study, we did not have any individual participant data on smoking habits and body mass index, and could therefore not include these factors as control variables in the analyses. We had, however, access to collateral data, which we have used to estimate age, gender and education standardized prevalence of smoking, overweight, and obesity among 20–59 year-old employees in Denmark, by type of employment contract (Hannerz et al. 2021). The estimated prevalence among people with fixed-term contracts were very similar to those among people with permanent contracts. It is therefore unlikely that the results of the present study have been influenced by differential prevalence of smoking, overweight and obesity.

We have not conducted any validation study of self-reported information on employment contract. We believe, however, that most (if not all) employees know if they have a permanent or temporary employment contract and that the question that was used to obtain the information in the present study is very easy to understand and difficult to misinterpret. Moreover, the question is not sensitive and it is not subject to recall bias. It should, however, be noted that our analysis do not account for time-variant unobservable characteristics that may have an impact on the results. It is, for example, possible that a person with fixed-term employment at baseline will become permanently employed or unemployed during the 5 year follow-up period. It is also possible that a person with permanent employment at baseline will become unemployed or shift to fixed-term contract position. Such transitions are probably associated with a bias toward unity.

In the present study, we used rate ratios of hospital treatment and redeemed prescriptions of drugs as proxy measures for underlying morbidity ratios. Hence, we need to consider the possibility of detection, prescription, and referral bias. In Denmark, all citizens are covered by a tax-funded health insurance, which enable them to consult a general practitioner and to receive psychiatric treatment free of charge, whenever it is needed. Since fixed-term and permanent employees have equal access to general practitioners as well as psychiatric hospitals and specialists, we do not think that the present study is subject any detection, prescription or referral bias of practical importance.

Psychiatric treatment is a rare event; hence, insufficient statistical power restricted the study of that outcome to a main effect only model. Psychotropic drugs include a few types that are used for disorders not expected to be associated with stressors like fixed-time contracts, e.g., psychostimulants and antidementia drugs. However, only a few promille of the cases were due to such drugs.

The underlying research hypothesis of the present project was that objective job insecurity may act as a stressor that increases a person’s vulnerability to mental ill health, without further specification. From this viewpoint, it may seem natural to include all types of mental disorders in the case definition of psychiatric hospital treatment. We chose, however, to exclude the vast majority (87%) of the diagnoses listed in the chapter on “mental and behavioral disorders” of the ICD-10 classification, and to focus solely on diagnoses that are labeled as mood, anxiety or stress-related disorders. We excluded F00–F09 “Organic mental disorders” because of their etiology in cerebral disease or brain injury, which make them quite irrelevant to the context of the present study; F60–F69 “Personality disorders”, F70–F79 “Mental retardation”, F80–F89 “Disorders of psychological development” and F90–F98 “Behavioral and emotional disorders with onset usually occurring in childhood and adolescence” because such disorders typically develop well before the entering of the labor market; somatoform disorders, firstly, because of an extraordinarily long expected duration between the onset of the complaints and the diagnosis (Herzog et al. 2018) and, secondly, because the labeling of such disorders as mental illnesses is controversial (Rief and Isaac 2007; Kroenke 2007); F10–F19 “Mental and behavioral disorders due to psychoactive substance use”, F42 “Obsessive–compulsive disorder” and F50–F59 “Behavioral syndromes associated with physiological disturbances and physical factors” because we wanted to keep our case definition simple and easy to communicate, which would not have been the case if we had included these diverse sets of behavioral disorders; and F20–F29 “Schizophrenia, schizotypal and delusional disorders” because they are associated with an extraordinarily high heritability (Hilker et al. 2018) and a low labor market attachment (Marwaha and Johnson 2004; Rinaldi et al. 2010), which make them quite irrelevant to the context of the present study. Here, it should be noted that the last mentioned category contains F25 “Schizoaffective disorders” and that manic, bipolar and depressive schizoaffective disorders thereby were excluded from our case definition.

We chose to base our case definition on diagnostic standard groupings at the two- or three-character level rather than on an ad hoc collection of four-digit level sub-categories, for several reasons. Firstly, because we wanted to decrease the probability that relevant cases were missed. Secondly, because the probability of misclassifications, i.e., false positive and false negative diagnoses, are likely to be higher at the four-character level than they are at the two and three-character level (Jensen et al. 2010). Thirdly, because a wider diagnostic category is less sensitive to random variation than a narrower diagnostic category.

Since (i) participants who received social security cash benefits, sickness absence benefits, psychotropic medicines or psychiatric hospital treatment within a 1-year period prior to baseline were excluded from the analysis and (ii) most of the mental disorders that are likely to depend on factors occurring before adulthood were excluded from the case definition, we do not believe that the study is subject to reverse causality bias of practical importance. The case definition included, however, phobic anxiety disorders (ICD-10: F40), which often manifest themselves already in childhood or adolescence (Kessler et al. 2007; Solmi et al. 2022). It is possible that some of the cases of phobic anxiety that were observed in the present study existed already at the start of the follow-up. It is also possible that that some people may be unable to obtain or hold a permanent employment position due to phobic anxiety disorders. Hence a possibility of reversed causation. To explore this possibility, we conducted a post hoc sensitivity analysis in which we excluded phobic anxiety disorders from the case definition. All other details of the analysis were the same as in the primary analysis of the psychiatric hospital treatments. In this post hoc sensitivity analysis, the concerned rate ratio was estimated at 1.42 (99.5% CI 1.06–1.91).

To further explore the possibility of reversed causation in the analysis of the hospital treatment data, we conducted a post hoc sensitivity analysis in which we extended the required period of “no psychiatric hospital treatment” from one to five-year prior to the baseline interview. All other details of the analysis were the same as in the primary analysis of the psychiatric hospital treatments. In this post hoc sensitivity analysis, the concerned rate ratio was estimated at 1.34 (99.5% CI 0.98–1.82).

In the analysis of psychotropic drugs, we aimed at estimating the association between our exposure variable and redeemed prescriptions for psychotropic medicine, and with such an aim, it made sense to include all types of psychotropic medicine in the case definition.

Two types of health selection bias need to be considered in the interpretation of the results. The first one concerns the possibility of bias due to health selection into a fixed-term or permanent employment position. It is, for example, possible that some people are unable to obtain or hold a permanent employment position due to lingering mental health problems. The second type of bias concerns health selection into the analysis. In our primary analysis, we included only DLFS participants with no social security cash benefits, no sickness absence benefits, no redeemed prescriptions for psychotropic medicines and no psychiatric hospital treatment during a whole year prior to the baseline interview. It was, moreover, required that they were full-time employees at the time of the interview. The purpose of the rigorous inclusion criteria was to counter potential bias from health selection into a fixed-term employment position. The consequence of the rigorous inclusion criteria is that the subset of fixed-term contract employees that was included in the primary analysis was far from representative of the full set of DLFS participants with a fixed-term contract position at baseline. It goes without saying that those who were permanently employed at baseline are more likely to have been permanently employed also prior to baseline and vice versa. Hence, if there are any health risks associated with not having a permanent employment contract then the fixed-term employees who were included in the primary analysis are likely to be more privilege and less vulnerable to the consequences of not having a permanent employment than the ones who were excluded. Seen from this perspective, selecting away cases 1 year—and especially 5 years—prior to baseline can be regarded as a very conservative approach underestimating possible effects of fixed-term contracts on depressive symptoms, as effects can have occurred before the follow-up period.

To shed some light on these health selection effects, we conducted two sensitivity analyses. In one of the analyses, we (i) removed the requirement of not receiving sickness benefits or social security cash benefits during a 1-year period prior to the baseline interview and (ii) removed all control variables except for gender, age, and education. We kept, however, the requirement of full-time employment at baseline and no redeemed prescriptions for psychotropic medicines and no psychiatric hospital treatment during a whole year prior to the baseline interview. The purpose of this analysis was to obtain an unbiased estimate of the rate ratio of psychotropic drug use between “a representative set of the DLFS participants with a full-time fixed-term contract position” and “a representative set of the DLFS participants with full-time permanent employment” after standardization for gender, age and education. The rate ratio in this analysis was estimated at 1.31 (99.5% CI 1.21–1.42). In another sensitivity analysis, we extended the required period of “no redeemed prescriptions for psychotropic medicines and no psychiatric hospital treatment” from 1 to 5 years prior to the baseline interview (on top of the rigorous inclusion criteria and potentially over-adjusted confounder control of the primary analysis). In this sensitivity analysis, the rate ratio of psychotropic drug use between employees with fixed-term vs. permanent employment contracts was estimated at 1.05 (99.5% CI 0.90—1.23). Further details about our pre-specified sensitivity analyses are given in the appendix.

### Generalizability

The results of the present paper should be seen in the light of specific conditions at the Danish labor market, which have been labeled flexicurity, a certain combination of low employment contract protection and generous compensation levels regarding unemployment benefits (Bredgaard and Madsen 2018; Madsen 2006, 2013). Effects of fixed-term contracts on health might be dependent on the welfare states’ employment protection regarding fixed-term and permanent contracts (Voßemer et al. 2018). This means that experienced job insecurity in fixed-term and permanent contracts could vary considerably between welfare state regimes making inference of study results across countries difficult.

### Conclusions

We know very little about the possible effects of contract type and mental health across countries. Increased cooperation between labor market and health researchers could contribute to shed more light into this question. The present study supports the hypothesis that employment in a fixed-term rather than permanent contract position is associated with an increased risk of developing mental health problems in Denmark. The results in themselves do not warrant specific interventions regarding fixed-term contracts as they can range from (a) restrictions in establishing fixed-term contracts over (b) improvements in working conditions for this group of workers to (c) specific health-related interventions. We can, however, conclude that the results of the study lend support to the necessity of the EU council directive 1999/70/EC of 28 June 1999 concerning the framework agreement on fixed-term work (The Council of the European Union 1999). The notably higher RR within transport and storage and to a lesser extent construction industries might warrant a particular focus on possible preventive efforts in these industries.

## Supplementary Information

Below is the link to the electronic supplementary material.

Supplementary file1 (DOCX 29 KB)
