# Supplementary material for: Mental illness rates among employees with fixed-term versus permanent employment contracts: a Danish cohort study
Source: Int Arch Occup Environ Health. 2022 Nov 22;96(3):451–62. doi: 10.1007/s00420-022-01936-7 (PMC9968265; doi:10.1007/s00420-022-01936-7)
Supplement: Supplementary file 1 — Supplementary file1 (DOCX 29 KB) [file 420_2022_1936_MOESM1_ESM.docx]

# Mental illness rates among employees with fixed-term versus permanent employment contracts: A Danish cohort study^1^

by Harald Hannerz, Fil.Lic, Hermann Burr, PhD^2^, Martin Lindhardt Nielsen, PhD, Anne Helene Garde, PhD, Mari-Ann Flyvholm, PhD

1. *Supplementary material*
2. *Correspondence to: Hermann Burr, Federal Institute for Occupational Safety and Health, BAuA, Nöldnerstr. 40–42, 10317 Berlin, Germany. [E-mail: burr.hermann@baua.bund.de].*

# Sensitivity Analyses

In accordance with our study protocol [Hannerz et al., 2021], we conducted a series of sensitivity analyses on the associations between fixed-term employment contracts and use of psychotropic drugs among employees in the Danish labor force. The purpose, methods and the results of the sensitivity analyses are given in the present appendix. The text that describes the purpose and methods of the analyses has been adapted (copied or slightly amended) from our study protocol, which contains the following copyright and license information:

“©Harald Hannerz, Hermann Burr, Helle Soll-Johanning, Martin Lindhardt Nielsen, Anne Helene Garde, Mari-Ann Flyvholm. Originally published in JMIR Research Protocols (http://www.researchprotocols.org), 05.02.2021. This is an open-access article distributed under the terms of the Creative Commons Attribution License (https://creativecommons.org/licenses/by/4.0/), which permits unrestricted use, distribution, and reproduction in any medium, provided the original work, first published in JMIR Research Protocols, is properly cited. The complete bibliographic information, a link to the original publication on http://www.researchprotocols.org, as well as this copyright and license information must be included.”

## Sensitivity Analysis 1: Exclusion of All Cases that Occurred Within 5 Years Preceding the Start of Follow-Up

In the primary analysis, we excluded all people who received psychiatric hospital treatment or redeemed a prescription for psychotropic drugs sometime during a 1-year period before the start of the follow-up. Hence, no known current cases of psychiatric treatment were included in the follow-up. It was, however, possible that people who received treatment more than 1 year before the follow-up would influence the analysis. To shed some light on this issue, we conducted a sensitivity analysis in which we excluded all people who received psychiatric hospital treatment or redeemed a prescription for psychotropic drugs sometime during a 5-year period before the start of the follow-up. This sensitivity analysis was based on data from the participants’ first interviews in the period 2005-2013. Moreover, it included only people who lived in Denmark throughout the concerned 5-year period. The statistical models and inclusion criteria were otherwise the same as in the primary analysis. The results of this sensitivity analysis are given in Table S1. According to the study protocol, the interpretation of the results would include the fact that approximately 20% of the population experiences mental health problems during their lifespan due to different causes; so the analysis may be over-adjusted. We note that the estimated RR in this sensitivity analysis is lower than the one obtained in the primary analysis, which weakens the finding of the primary analysis.

Table S1. Rate ratio (RR) with 99.5% confidence interval (CI) for incident use of psychotropic drugs, as a function of type of employment contract among full-time employees in Denmark 2005 - 2013

| Type of employment contract | Persons | Person years | Cases | RR^a^ | 99.5% CI |
| --- | --- | --- | --- | --- | --- |
| Fixed-term | 4852 | 18 614 | 427 | 1.05 | 0.90 - 1.23 |
| Permanent | 70 271 | 278 676 | 5785 | 1.00 | - |

^a^Adjusted for age, gender, industrial sector, nighttime work, education, calendar year, disposable family income and state educational grants, unemployment benefits and maternity/paternity benefits within one-year prior to baseline

## Sensitivity Analysis 2: Relapse Rate Ratios

To further examine the possible influence of former cases of psychiatric treatment on the association between fixed-term contract and psychotropic drug usage, we estimated relapse RRs among the participants who were excluded from sensitivity analysis 1 due to psychiatric hospital treatment or redeemed prescription for psychotropic drugs sometime between 1 and 5 years before the start of follow-up. Current cases, that is, people who received treatment within a 1-year period before the start of follow-up were not included in the analysis. The statistical model was otherwise the same as in sensitivity analysis 1. The results are given in Table S2. We note that the estimated RR for relapsed use of psychotropic drugs is the same as the RR for incident use.

Table S2. Rate ratio (RR) with 99.5% confidence interval (CI) for relapsed use of psychotropic drugs, as a function of type of employment contract among full-time employees with a past record of psychiatric treatment

| Type of employment contract | Persons | Person years | Cases | RR^a^ | 99.5% CI |
| --- | --- | --- | --- | --- | --- |
| Fixed-term | 429 | 1410 | 145 | 1.12 | 0.86 - 1.45 |
| Permanent | 6155 | 20 227 | 1971 | 1.00 | - |

^a^Adjusted for age, gender, industrial sector, nighttime work, education, calendar year, disposable family income and state educational grants, unemployment benefits and maternity/paternity benefits within one-year prior to baseline

## Sensitivity Analysis 3: Long-term Exposure versus Exposure at a Single Time Point

In the primary analysis, we regarded the contrast full-time fixed-term contract versus full-time permanent contract with the exposure categories defined at a single time point (the first interview). To find out if the strength of the association would increase if we defined the exposure categories on long-term exposure instead of exposure at a single time point, we conducted a sensitivity analysis, in which we only included people whose exposure was table over time. In this particular analysis, we included participants who (i) participated in more than one interview, (pp) were aged between 20 and 59 years during their last interview, and (iii) belonged to the same exposure category in all of their interview rounds. The follow-up of the included participants commenced 6 weeks after their last interview. The statistical models and inclusion criteria were otherwise the same as in the primary analysis. The results are given in Table S3. We note that the estimated RR increases when the analysis is restricted to employees with stable exposure, which strengthens the finding of the primary analysis.

Table S3. Rate ratio (RR) with 99.5% confidence interval (CI) for incident use of psychotropic drugs among full-time employees in Denmark 2001 – 2013, with stable exposure to either fixed-term or permanent employment contracts

| Type of employment contract | Persons | Person years | Cases | RR^a^ | 99.5% CI |
| --- | --- | --- | --- | --- | --- |
| Fixed-term | 1889 | 6881 | 197 | 1.16 | 0.93 - 1.43 |
| Permanent | 70 662 | 265 713 | 6899 | 1.00 | - |

^a^Adjusted for age, gender, industrial sector, nighttime work, education, calendar year, disposable family income and state educational grants, unemployment benefits and maternity/paternity benefits within one-year prior to baseline

## Sensitivity Analysis 4: Minimally Adjusted Rate Ratios

In the primary analyses, we excluded all people who received sickness benefits or social security cash benefits during a 1-year period before the baseline interview. Moreover, we controlled for disposable family income as well as a series of other covariates. It is possible that the rigorous inclusion criteria and the many control variables would lead to overly conservative estimates. To shed light on this issue, we conducted a sensitivity analysis in which we (i) removed the requirement of not receiving welfare benefits (other than holiday allowance, unemployment benefits, maternity/paternity benefits or state educational grants) during a one-year period prior to the baseline interview and (ii) removed all control variables except for gender, age, and education. The methods were otherwise the same as in the primary analyses. The results are given in Table S4. We note that the estimated RR increases (from 1.12 to 1.31) when we only control for gender, age and education, which strengthens the finding of the primary analysis.

Table S4. Rate ratio (RR) with 99.5% confidence interval (CI) for incident use of psychotropic drugs, as a function of type of employment contract among full-time employees in Denmark 2001 - 2013

| Type of employment contract | Persons | Person years | Cases | RR^a^ | 99.5% CI |
| --- | --- | --- | --- | --- | --- |
| Fixed-term | 10 188 | 39 798 | 1483 | 1.31 | 1.21 - 1.42 |
| Permanent | 114 942 | 465 560 | 13 700 | 1.00 | - |

^a^Adjusted for age, gender and education level

## Sensitivity Analysis 5: Reason for Being on a Fixed-Term Contract

All EU-Labor Force Survey participants with a fixed-term contract are asked for the reason of having a fixed-term contract. Their answers are categorized as follows:

1. It is a contract covering a period of training (apprentices, trainees, research assistants, etc.)

2. Person could not find a permanent job

3. Person did not want a permanent job

4. It is a contract for a probationary period

We wanted to know whether the risk of developing mental health illnesses among employees with a fixed-term contract depends on the reason for being on a fixed-term contract. To answer this question, we estimated incidence RRs for redeemed prescriptions of psychotropic drugs as a function of the reason for being on a fixed-term contract. Participants who did not want a permanent job (category 3) would serve as the reference group. We included all employees on fixed-term contracts, who fulfilled inclusion criteria 1-5, as listed in the method section. The analysis was initially conducted only with full-time employees (≥32 h a week) and then with all employees regardless of weekly working hours. The statistical model and follow-up periods were the same as in the primary analysis. The results are given in the tables S5A and S5B. We note that the estimated rates were lower, albeit not significant, among the employees who did not want a permanent job than they were among the employees in the other categories. The results of present sensitivity analysis thereby suggest that the association between fixed-term contracts and mental ill health might depend on the reason for having a fixed-term contract.

Table S5A. Rate ratio (RR) with 99.5% confidence interval (CI) for incident use of psychotropic drugs, as a function of type of employment contract among full-time employees in Denmark 2001 - 2013

| Reason for Being on a Fixed-Term Contract | Persons | Person years | Cases | RR^a^ | 99.5% CI |
| --- | --- | --- | --- | --- | --- |
| Training period | 2542 | 9525 | 234 | 1.19 | 0.86 - 1.64 |
| Could not find a permanent job | 3472 | 13 752 | 485 | 1.07 | 0.81 - 1.4 |
| Probationary period | 132 | 451 | 24 | 1.82 | 0.97 - 3.41 |
| Did not want a permanent job | 1222 | 5097 | 147 | 1.00 | - |

^a^Adjusted for age, gender, industrial sector, nighttime work, education, calendar year, disposable family income and state educational grants, unemployment benefits and maternity/paternity benefits within one-year prior to baseline

Table S5B. Rate ratio (RR) with 99.5% confidence interval (CI) for incident use of psychotropic drugs, as a function of type of employment contract among part-time and full-time employees in Denmark 2001 - 2013

| Reason for Being on a Fixed-Term Contract | Persons | Person years | Cases | RR^a^ | 99.5% CI |
| --- | --- | --- | --- | --- | --- |
| Training period | 2707 | 10 069 | 256 | 1.22 | 0.93 - 1.61 |
| Could not find a permanent job | 5191 | 20 470 | 745 | 1.13 | 0.91 - 1.41 |
| Probationary period | 172 | 580 | 28 | 1.70 | 0.96 - 3.01 |
| Did not want a permanent job | 1978 | 8286 | 230 | 1.00 | - |

^a^Adjusted for age, gender, industrial sector, nighttime work, education, calendar year, disposable family income and state educational grants, unemployment benefits and maternity/paternity benefits within one-year prior to baseline

## Sensitivity Analysis 6: Stratification by Industry

We know that the prevalence of fixed-term contracts in the Nordic countries depends on the industry sector [Rasmussen et al., 2019], and that the rates of mood disorders in the general working population of Denmark depend on the industry sector [Hannerz et al., 2009]. It is possible that the effect of fixed-term contract positions on mental health illnesses also depends on the industry. We therefore conducted a sensitivity analysis in which we stratified the results of the comparison between employees with a fixed-term and a permanent contract by the industry sector. The inclusion criteria and covariates were the same as in the primary analysis. Reasons why the association between fixed-term contracts and mental health illnesses might depend on industry could be, first, that chances for reemployment may depend on the industry sector and, second, that expectations regarding a fixed-term versus permanent contract may depend on the industry. Another reason for stratifying by the industry sector was that the social partners might be interested in seeing the association between fixed-term contracts and mental health illnesses in their own industry sector. The results of this sensitivity analysis suggest that the effect of fixed-term employment might be especially high in the transporting and storage industry (Table S6).

Table S6. Rate ratio (RR) with 99.5% confidence interval (CI) for incident use of psychotropic drugs as a function of type of employment contract, stratified by industrial sector, among full-time employees in Denmark 2001 – 2013

| Industrial group | Type of employment contract | Persons | Person years | Cases | RR^a^ | 99.5% CI |
| --- | --- | --- | --- | --- | --- | --- |
| Agriculture, forestry, hunting, and fishing | Fixed-term | 153 | 604 | 10 | 0.81 | 0.32 - 2.03 |
|  | Permanent | 1447 | 6063 | 121 | 1.00 | - |
| Manufacturing, mining, and quarrying | Fixed-term | 662 | 2674 | 75 | 1.07 | 0.77 - 1.50 |
|  | Permanent | 17 558 | 73 334 | 1870 | 1.00 | - |
| Construction | Fixed-term | 444 | 1747 | 41 | 1.23 | 0.78 - 1.95 |
|  | Permanent | 5404 | 22 576 | 474 | 1.00 | - |
| Wholesale, retail and repair of motor vehicles | Fixed-term | 864 | 3254 | 89 | 1.21 | 0.88 - 1.65 |
|  | Permanent | 12 880 | 52 574 | 1240 | 1.00 | - |
| Transporting and storage | Fixed-term | 184 | 722 | 34 | 1.87 | 1.14 - 3.07 |
|  | Permanent | 6040 | 24 963 | 652 | 1.00 | - |
| Accommodation and food service activities | Fixed-term | 222 | 833 | 21 | 1.01 | 0.52 - 1.94 |
|  | Permanent | 1349 | 5260 | 139 | 1.00 | - |
| Human health and social work activities | Fixed-term | 1394 | 5505 | 196 | 1.04 | 0.84 - 1.29 |
|  | Permanent | 15 931 | 62 808 | 2154 | 1.00 | - |
| Other industries | Fixed-term | 2905 | 11 382 | 363 | 1.14 | 0.97 - 1.34 |
|  | Permanent | 36 937 | 147 963 | 3889 | 1.00 | - |
| Unstated | Fixed-term | 632 | 2520 | 74 | 0.97 | 0.66 - 1.44 |
|  | Permanent | 1495 | 5949 | 174 | 1.00 | - |

^a^Adjusted for age, gender, nighttime work, education, calendar year, disposable family income and state educational grants, unemployment benefits and maternity/paternity benefits within one-year prior to baseline.

# References

Hannerz H, Burr H, Soll-Johanning H, Nielsen ML, Garde AH, Flyvholm MA. Prospective Associations Between Fixed-Term Contract Positions and Mental Illness Rates in Denmark's General Workforce: Protocol for a Cohort Study. JMIR Res Protoc. 2021 Feb 5;10(2):e24392. doi: 10.2196/24392

Hannerz H, Tüchsen F, Pedersen BH, Dyreborg J, Rugulies R, Albertsen K. Work-relatedness of mood disorders in Denmark. Scand J Work Environ Health. 2009;35(4):294-300.

Rasmussen S, Nätti J, Larsen TP, Ilsøe A, Garde AH. Nonstandard employment in the Nordics – toward precarious work? Nordic J Working Life Stud 2019 Jun 01;9(S6)
